# Sex differences in the relative heterogeneity of frailty in relation to age, frailty, health protection, and five‐year mortality

**DOI:** 10.1002/agm2.12090

**Published:** 2019-12-01

**Authors:** Zhan Yang, Chunxiu Wang, Zhe Tang, Xiaowei Song

**Affiliations:** ^1^ Department of Science in Biology Crandall University Moncton NB Canada; ^2^ Department of Evidence‐based Medicine Beijing Xuanwu Hospital Capital Medical University Beijing China; ^3^ Health Sciences and Innovation Surrey Memorial Hospital Fraser Health Authority Surrey BC Canada; ^4^ Department of Biomedical Physiology and Kinesiology Simon Fraser University Burnaby BC Canada

**Keywords:** aging, coefficient of variation, frailty index, protection factors, sex difference

## Abstract

**Background:**

Previous studies have suggested that the relative heterogeneity of frailty declines with increases in age and the level of the frailty index (FI). In this study, we investigated the sex difference in the relative heterogeneity of frailty and its response to health‐protective factors, in a Chinese community sample.

**Methods:**

Data used for this secondary analysis were obtained from the Beijing Longitudinal Study of Aging that involved 3257 community‐dwelling Chinese people aged 55 years and older at baseline. An FI was constructed for each indicial using 35 variables assessing health‐related problems. A protection index (PI) consisting of 27 variables assessing lifestyle and social engagement was also built. The relative heterogeneity of frailty, as measured by the coefficient of variation (CV) of the FI, was calculated as the ratio of the standard deviation to the mean FI for different age, FI, and PI groups, and for the five‐year survival status.

**Results:**

The CV decreased with the increase in age (*F* = 20.60, *P* = .006) and the FI (*F* = 57.59, *P* = .001), consistent in both sexes. In each age group, the CV was higher in men than in women (*t* = 3.25, *P* = .018). A great level of protection was associated with a significantly reduced mortality, and an increased CV (*t* = 2.91, *P* = .027).

**Conclusions:**

Our data demonstrate that a gender difference exists in the relative heterogeneity of frailty, which is negatively related to age and frailty as well as positively associated with health protection and the five‐year survival.

## 1 INTRODUCTION

Frailty is a dynamic and stochastic state,^1^, ^2^, ^3^ which can vary substantially in older adults. Frailty heterogeneity has not only been associated with intrinsic factors such as chronologic age, sex, genes, physiological and psychological factors,^4^, ^5^ but also associated with extrinsic factors such as lifestyle, environment, socioeconomic levels, and social support factors.^6^, ^7^ The frailty index (FI) based on the accumulation of health deficits has been used to assess frailty in multiple studies.^8^, ^9^

As people age they have cumulative deficits, which are accompanied by decreased redundancy of a body system over the life span,^10^, ^11^ leading to an irreversible process of deterioration in the body system and, finally death. During the deterioration process, older adults show highly heterogeneous health status. When the body system reaches its redundancy exhaustion (i.e. an empirical limit with an FI value around 0.7) as detected in several studies,^12^, ^13^, ^14^ maintenance of its homeostasis becomes dysfunctional in response to stressors, leading to catastrophic health transition.^15^ As people get closer to the end of life, a single extra deficit can cause the body system to fail, leaving the individual with little chance of survival.

Increased health vulnerability in older adults can be well represented by a decline in relative heterogeneity of FI. Such frailty heterogeneity can be measured by the coefficient of variation (CV), i.e. the ratio of the standard deviation to the mean FI. It declines with an increase in age, which is well applied to Ashby's law of “requisite variety”^16^ and indicates that as a complicated system ages, it loses variety in the response repertoire and is accompanied by decreasing capability of perceiving disturbance, so that the system is incapable of responding to disturbances and maintaining its integrity.^17^

Frailty may be treatable, benefited from numerous geriatric interventions.^18^, ^19^, ^20^, ^21^ Even though deterioration and death generally dominate the process of aging, short‐term stabilization and also improvement in health status can occur in some individuals.^22^ Using the Beijing Longitudinal Study of Aging data, we have shown that extrinsic factors related to lifestyle, behavioral, social and environmental factors can exert positive effects of protection on health outcomes.^23^ Lower levels of mortality and health worsening have been reported in people with a higher summative result of multiple protective factors. It is suggested that protective factors might slow down the progress of losing variety in response repertoire with aging, making the variety of responses outnumber the variety of disturbances and thus keeping people alive and healthier longer.

In this study we investigated the age and sex differences in the relative heterogeneity of frailty and examined their relations with the level of health protection. To do so, we compared men and women of different age and frailty groups and with different five‐year mortality outcomes. Given the extent of health changes, we separately studied subjects who were aged 55‐64 years and those aged 65+ years in a well‐established Chinese community sample.

## 2 METHODS

### 2.1 Participants and data

As described elsewhere, the Beijing Longitudinal Study of Aging is a prospective cohort study of 3257 community‐dwelling Chinese population aged 55 years and older at baseline. The geographic distribution, economic status, age, and education of the sample represent the older population of Beijing, as obtained from the Fourth National Census Data.^24^ As described elsewhere,^25^ the cohort was assembled in 1992; the response rate was 91.2%; participants were followed every two to three years. At the time of the 1997 survey, 784 subjects (24.1%) had died, 430 were lost to follow‐up. The study was based on self‐reporting information that covered demographic characteristics, socioeconomic status, activities of daily living, lifestyle, physical health, psychological and self‐rated health, medical conditions, cognitive status, and the use of health care services. Trained interviewers, mostly nurses or physicians, administered a standard questionnaire at the respondent's home; where available, medical records were used to verify the presence of disease. Depressive symptoms were evaluated using the Center for Epidemiologic Studies Depression Scale (CES‐D), and cognitive function was assessed using the Mini‐Mental State Examination (MMSE).

As with previous studies,^14^, ^23^, ^26^, ^27^ for this analysis, variables from the baseline dataset were retrieved. In this study, the widely accepted age of 65 years as the threshold of older verses later middle‐aged adults^28^, ^29^, ^30^ was used as the cut‐off age. Five‐year survival outcomes were evaluated. Survival status was determined through interviews with surviving household members and neighbors and verified by death certificates and/or local police register records. Vital status was known for 92.1% of the participants, with censoring for dates of death or dropout. Data of the subjects with missing survival information (8.4%) were excluded from survival‐related analysis only.

### 2.2 Frailty index

A frailty index (FI) was constructed using the baseline survey data (1992) for each participant as described elsewhere.^14^, ^23^, ^26^, ^27^ Each variable used in the FI satisfied the criteria of being associated with health status, accumulating with age, not saturating (i.e. not becoming present in >80% of people), and having >1% prevalence and <5% missing, and covering several systems.^31^ In total 35 variables were used, which included diseases (n = 8), symptoms (n = 7), psychological problems (n = 5), basic and Instrumental Activities of Daily Living (ADL and IADL) disabilities (n = 14), the MMSE total score.^14^ The variables were each coded into a value between 0 and 1; the coded values were then summed and divided by 35 (the ratio of deficits present). This operation yielded an FI ranging from a theoretical minimum of 0 (no deficits present) to a possible maximum of 1.0 (all deficits present), with higher FI values representing a higher level of frailty, representing worse health and greater vulnerability to adverse outcomes. The maximum number of missing value with any individual in the sample was 1, which was replaced using the non‐missing mean.

### 2.3 Relative heterogeneity of frailty

The relative heterogeneity was calculated as the coefficient of variation (CV) of the frailty index (FI) based on the equation: *ν* = *σ*/〈*f*〉, where *ν* is coefficient of variation, 〈*f*〉 represents the mean FI and *σ* is its standard deviation for any given age group.^22^ The coefficient of variation at a given level of FI 〈*f*〉 is described by a power‐law formula: *ν* = *A*/〈*f*〉*^α^*, where the parameters *A* and *α*, are fitted to the observed data.^22^

### 2.4 Protection Index

A Protection Index (PI) was constructed as the sum of 27 extrinsic factors, representing a comparably complete version of health protection assessment than previously reported.^23^ Each of the items was coded in a reverse manner as with the deficits coding. For instance, for the PI, 1 = being married; satisfied with house condition; someone help housework; having someone counts for help; family or friend to count on help; financial help acquired; visit friend or relatives; traveling; help your relative to do housework; physical or outdoor leisure activities; sedentary leisure activities; not affected by the relationship with your spouse; not affected by the relationship with your neighbor; not affected by the relationship with your children; feel useful; feel life meaningful; like to make friends; like to be together; safe living environment; quiet living environment; not affected by your financial status; having a high (>9 years) level of education; eat vegetables regularly; eat fruit regularly; eat seafood regularly; do not smoke; do not drink alcohol regularly. The coded values were summed and divided by 27 to yield a PI ranging from a theoretical minimum of 0 (no protection factor) to a maximum of 1.0 (the maximum number of protection factors), with higher PI values representing better protection.

### 2.5 Statistical analysis

Sample characteristics were described using means and standard deviations for interval variables and percentages for the categorical variables, with differences tested using analysis of variance (ANOVA) and Chi‐square (*Χ* ^2^) respectively. The attributable risk (AR) for the five‐year mortality was calculated for each protective factor as the fraction to the proportion of the risk among the exposed population lacking that protective factor that could be attributed to the exposure.^32^ Five‐year mortality rates were compared between men and women using Student *t* tests. Multivariable regression analysis was used to examine the relationship between the coefficient of variation (CV) with age and the FI. Based on the PI values, subjects were categorized into three groups (tertiles), with lower (1st tertile), intermediate (2nd tertile) and higher (3rd tertile) levels of protection. Age trajectories of the FI and its coefficient of variation were compared for sex and protection levels. Data analyses were performed using SPSS v21.

## 3 RESULTS

Women were slightly older and had less education than men (Table 1). Compared with women, men were more likely to be married and engaged in intellectual occupation especially after 65 years of age. In both men and women, most deficits were associated with an increased risk of mortality by five years. On average, women who were aged 55‐64 years appeared to be frailer (FI = 0.09 ± 0.07) and had more protective factors (PI = 0.71 ± 0.09) than men (FI = 0.08 ± 0.07, PI = 0.68 ± 0.10). Women aged 65+ years also tended to be frailer than men, but with no significant sex difference in PI (0.65 ± 0.11 vs 0.66 ± 0.11, *F* = 1.9, *P* = .169). The relative frailty heterogeneity, measured by the coefficient of variation CV of the FI, was higher in men than women in both age groups coefficient of variation (Table 1).

**Table 1:** Characteristics of the sample as separated by sex for the younger (<65 y) and older (>65 y) groups

	55‐64 y old				>65 y old			
	Men	Women	*F*	*P*‐value	Men	Women	*F*	*P*‐value
N	482	557			1111	1107		
Age	59.9 ± 4.2	59.8 ± 2.8	0.22	.641	74.6 ± 6.3	75.3 ± 6.7	4.87	.027
Education ≥ 9 y (%)	23.2	12.6	20.36	≤.001	15	5.1	60.98	≤.001
Death rate (%)	9.5	6.1	4.30	.038	33.8	30.7	2.35	.126
Marriage status (married) (%)	92.7	84.2	18.05	≤.001	69.5	40.1	193.23	≤.001
Occupation (Intellectual) (%)	27.2	24.3	33.32	≤.001	22.1	12.3	139.88	≤.001
MMSE score (/30)	25.7 ± 2.8	23.7 ± 3.6	63.5	<.001	24.1 ± 3.8	20.7 ± 4.4	231.02	≤.001
Number of deficits, mean ± SD (/35)	2.7 ± 2.5	3.2 ± 2.3	12.91	<.001	4.4 ± 3.4	5.7 ± 4.0	71.20	≤.001
Frailty Index, mean ± SD (higher is worse)	0.08 ± 0.07	0.09 ± 0.07	12.91	<.001	0.12 ± 0.10	0.16 ± 0.12	71.20	≤.001
Coefficient of variation (for FI)	0.94	0.72			0.78	0.71		
Number of protective factors, mean ± SD (/27)	18.3 ± 2.7	19.1 ± 2.6	20.49	<.001	17.5 ± 3.1	17.7 ± 3.1	1.89	.169
Protection index, mean ± SD (higher is better)	0.68 ± 0.10	0.71 ± 0.09	20.49	<.001	0.65 ± 0.11	0.66 ± 0.11	1.89	.169

Considering individually the factors that make up the PI, in both 55‐64 and 65+ age groups and especially the former, lack of protective factors often showed a higher risk of death in men than in women (Table 2). For the 55‐64 year‐old group, lack of many protective factors appeared to be more lethal for men than for women. This also occurred in the 65+ age group: lack of protective factors often correlated with a higher risk of death in men than in women. Notably, several protective factors, when considered individually, seemed to have promoted mortality in the 65+ group (i.e. negative attributable risk) after 5 years. This was especially true regarding women (Table 2). Such variables included several social support, life control factors.

**Table 2:** Absence of the protective factors and the associated attributable risks (AR) for the 5‐y mortality, by sex for the younger (<65 y) and older (>65 y) groups

N	55‐64 y‐olds				≥65 y‐olds			
	Men 482	Women 557	*χ* ^2^	*P*‐value	Men 1111	Women 1107	*χ* ^2^	*P*‐value
	% Present (AR)	% Present (AR)			% Present (AR)	% Present (AR)		
Living situation								
Not currently married	7.3 (0.68)	15.8 (0.12)	0.0	.828	30.5 (0.40)	59.9 (0.45)	69.0	<.001
Unsatisfied house condition	17.8 (−0.40)	16.9 (−0.15)	0.2	.640	16.7 (−0.81)	13.8 (−0.06)	3.6	.057
Social support								
No one help housework	11.6 (0.12)	30.5 (−0.05)	54.2	<.001	14.9 (−0.08)	26.2 (−1.43)	43.8	<.001
No one to count on for help	31.7 (0.08)	31.8 (−0.03)	0.0	.876	27.1 (−0.19)	23.3 (0.00)	2.0	.157
No family or friend to count on help	34.6 (0.25)	33.8 (−0.03)	0.8	.406	33.7 (−0.19)	34.4 (0.00)	0.7	.371
Financial help acquired	35.3 (0.23)	49.4 (0.32)	21.0	<.001	54.5 (0.40)	81.7 (0.36)	188.7	<.001
Social engagement and leisure								
Do not visit friends or relatives	95.2 (0.56)	89.0(−0.40)	13.3	<.001	93.9 (0.04)	91.4(0.08)	4.9	.026
No traveling	95.0 (1.00)	93.7 (1.00)	0.6	.427	97.2 (0.91)	98.9 (0.73)	8.5	.004
Do not help your relative to do housework	48.5 (0.39)	28.7 (0.55)	43.1	<.001	64.5 (0.49)	50.0 (0.54)	48.2	<.001
No physical or outdoor leisure activities	29.7 (0.34)	25.5 (−0.60)	2.3	.133	22.2 (0.35)	36.6 (0.45)	55.0	<.001
No sedentary leisure activities	4.4 (0.63)	2.5 (0.73)	2.7	.101	8.4 (0.47)	10.6 (0.44)	3.1	.077
Empowerment, life control								
Bad relationship with spouse	1.2 (0.73)	1.4 (0.47)	4.5	.034	1.4 ( −0.25)	1.4 (−0.62)	0.0	.856
Bad relationship with neighbors	1.5 (0.00)	1.1 (0.00)	0.3	.585	1.0(0.27)	0.9 (−0.51)	0.0	.8
Bad relationship with children	7.3 (0.20)	6.1 (0.33)	0.6	.450	6.2 (−0.37)	6.1(−0.20)	0.0	.859
Feel useless	40.5(0.34)	58.0(0.33)	31.3	<.001	56.2 (0.48)	70.1 (0.34)	79.3	<.001
Feel life meaningless	16.2 (0.48)	20.3 (0.52)	4.5	.107	18.2 (0.35)	24.5 (0.21)	16.0	<.001
Do not like making friends	16.4 (−0.41)	16.2 (0.11)	0.0	.876	21.7 (−0.12)	16.4 (0.04)	9.8	.002
Like to be alone	32.6 (0.08)	32.3 (−0.35)	0.5	.793	31.3 (0.05)	29.1 (−0.10)	1.4	.497
Unsafe living environment	1.0 (0.00)	1.3 (0.00)	0.1	.741	1.3 (0.35)	0.9 (0.01)	0.7	.417
Noisy living environment	42.9 (−0.56)	43.4 (−0.84)	0.0	.871	32.5 (−0.42)	26.7 (−0.15)	8.9	.003
Socio‐economic status								
Affected by financial status	2.1 (0.54)	2.0 (0.34)	0.0	.905	2.1 (0.50)	1.9 (0.21)	0.1	.761
Low education (<9 y)	76.8 (0.41)	87.4 (0.33)	20.4	<.001	85.0 (0.44)	94.9 (0.72)	61.0	<.001
Life style								
Do not eat vegetable regularly	2.9 (0.69)	2.5 (0.73)	0.2	.698	2.8 (0.40)	3.3 (0.38)	0.6	.451
Do not eat fruit regularly	45.6 (0.23)	45.4 (0.34)	0.0	.943	54.3 (0.31)	53.7 (0.27)	0.1	.771
Do not eat seafood regularly	62.7 (0.59)	63.4 (0.55)	0.1	.811	73.7 (0.46)	78.1 (0.22)	5.9	.015
Have smoking habit	51.0 (0.33)	15.3 (0.93)	152.4	<.001	40.2 (−0.12)	15.6 (0.26)	166.7	<.001
Regular alcohol drinker	40.0 (−0.25)	5.9 (−0.01)	9.4	.002	34.4 (−0.21)	6.7 (0.10)	59.4	<.001

Considering deficits collectively, the mean FI increased with age (Table 3a); the mean PI declined at advanced ages (Table 3b). Significant sex difference in FI was found in all age groups except the 55‐59 year‐old group (Table 3a), with women being frailer than men (*t* = 5.20, *P* = .002). The sex differences in the PI were chiefly showed in the 55‐64 year‐old group (Table 3b), while no significant sex differences existed in the 65+ age group.

**Table 3:** (a) The mean level of the Frailty Index (FI) by sex and age group. (b) The mean level of the Protection Index (PI) by sex and age group

Age group (y)	Men	N	Women	N	*F*	*P*‐value
(a)						
55‐59	0.07 ± 0.07	226	0.08 ± 0.06	248	2.04	.154
60‐64	0.08 ± 0.08	256	0.10 ± 0.07	309	10.99	.001
65‐69	0.10 ± 0.08	282	0.13 ± 0.10	274	16.70	<.001
70‐74	0.11 ± 0.09	302	0.14 ± 0.11	251	12.99	<.001
75‐79	0.13 ± 0.10	260	0.15 ± 0.10	260	9.82	.002
80‐84	0.16 ± 0.11	191	0.21 ± 0.13	223	17.74	<.001
85+	0.19 ± 0.11	76	0.24 ± 0.11	99	7.08	.009
(b)						
55‐59	0.68 ± 0.11	226	0.71 ± 0.08	248	18.14	<.001
60‐64	0.68 ± 0.10	256	0.70 ± 0.09	309	4.96	.026
65‐69	0.68 ± 0.10	282	0.69 ± 0.10	274	2.98	.085
70‐74	0.66 ± 0.11	302	0.68 ± 0.10	251	4.19	.041
75‐79	0.64 ± 0.11	260	0.65 ± 0.11	260	0.14	.708
80‐84	0.63 ± 0.12	191	0.63 ± 0.12	223	0.03	.871
85+	0.57 ± 0.14	76	0.59 ± 0.13	99	0.37	.543

**Figure 1:**
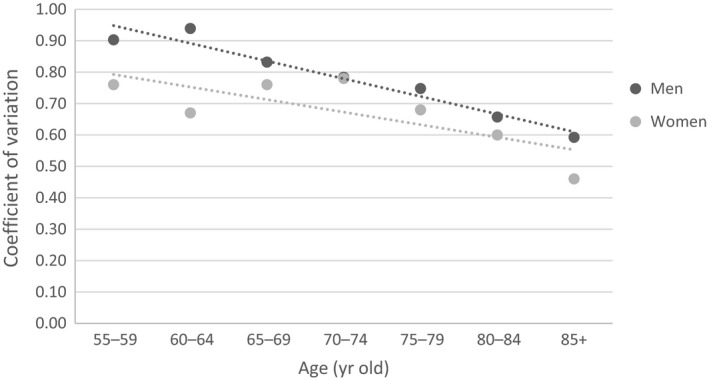
Sex difference of the relative heterogeneity of deficit accumulation based frailty index (i.e. the coefficient of variation of the frailty index) in relation to age group

**Figure 2:**
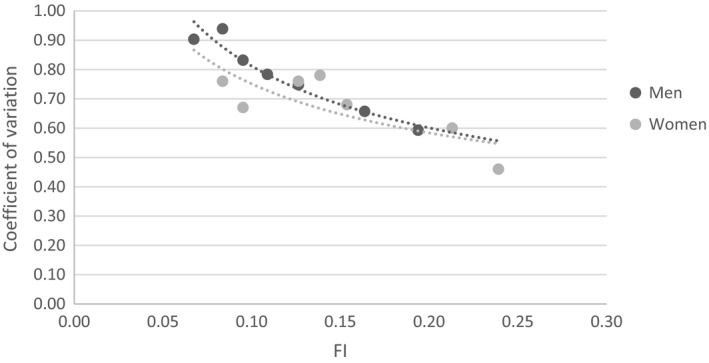
Sex difference of the relative heterogeneity of deficit accumulation based frailty index (i.e. the coefficient of variation of the frailty index) in relation to the mean level of the frailty index

**Figure 3:**
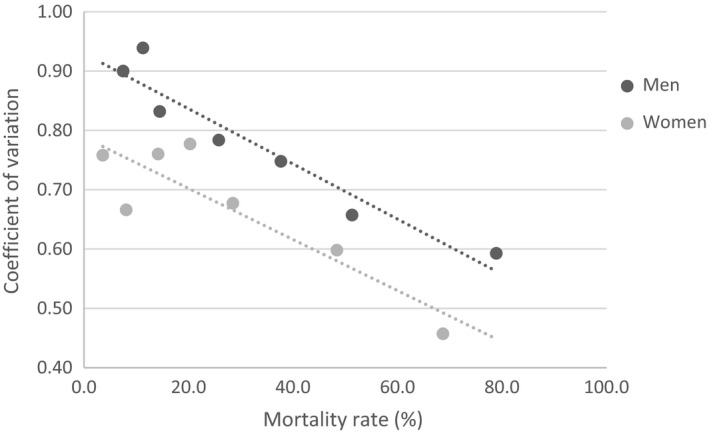
Sex difference of the relative heterogeneity of deficit accumulation based frailty index (i.e. the coefficient of variation of the frailty index) in relation to the five‐year mortality rate

The decline of the coefficient of variation (CV) of frailty was greater in men than in women (*t* = 3.24, *P* = .018; Figure 1). The relative frailty heterogeneity also decreased with an increase of the FI, similarly in both men and women (Figure 2). The relationship of coefficient of variation, *ν*, with the FI could be described by a power‐law formula: *ν* = *A*/〈*f* *^α^*, where the parameters: ln(*A*) = −1.24 (95% CI = −1.50, −0.98) and *α* = −0.45 (95% CI = −0.57, −0.33) in men; ln(*A*) = −1.13 (95% CI = −1.85, −0.40) and *α* = −0.37 (95% CI = −0.73, −0.00) in women. The coefficient of variation (CV) was negatively related to mortality in both men and women, such that a higher level of the relative heterogeneity of frailty was related to a lower level of five‐year mortality (Figure 3).

Possessing more protection factors (i.e. a higher level of the PI) was associated with a lower level of mortality in both men and women (*t* = 4.25 vs *t* = 3.26, *P*'s ≤ .017; Table 2). Similarly, a greater level of the PI was associated with a higher level of the relative heterogeneity of frailty as measured by the CV (*t* = 2.91, *P* = .027) in the sample.

## 4 DISCUSSION

In the previous studies, women in comparison with men have lower self‐rating on health and tend to accumulate more deficits.^33^, ^34^, ^35^, ^36^ However, women appear to tolerate more deficits and have greater capability in compensating for adverse outcomes accompanied by lower mortality rate.^14^ In this study we evaluated the relative heterogeneity of frailty in a Chinese community sample, applying the deficit accumulation based frailty index (FI) approach. We studied the sex differences of the relative heterogeneity of frailty in relation to age, the FI, the protection index (PI), and the five‐year mortality. By constructing an improved version of the PI that consisted of a complete set of the protective factors available in the dataset, we further examined the impact of health protection on the relative heterogeneity of frailty, providing the first investigation of this research line.

Our data confirmed that the relative heterogeneity measured by the coefficient of variation declined with increased age and FI, consistent with the previous reports.^22^, ^33^ Our data also confirmed that higher mortality rates were associated with lower levels of the relative heterogeneity of frailty. This study further extended the frailty heterogeneity analysis to include people of late middle age. By examining the effect of health‐protective factors on the relative heterogeneity of frailty, this paper provides the first evidence of the positive impact of health protection on health heterogeneity, suggesting the interplay between intrinsic and extrinsic factors in determining health outcomes. Moreover, comparing men and women in the analyses reveals the sex differences of the relative heterogeneity of frailty in relation to age, frailty, health protection, and the five‐year survival.

Consistent with previous studies, this dataset showed women have a higher level of deficit accumulation and lower death rates compared to men. Meanwhile, women, especially those of a more advanced age, were more likely to have lower cognitive performance and engage less in intellectual occupations. Similar results have been shown in different datasets,^34^, ^35^, ^36^ and are confirmed in the Chinese sample.

As expected, the majority of the protective factors were individually associated with a decreased risk of death, meaning that a lack of protective factors increased the individual's risk of mortality (Table 2). Meanwhile, a few variables, e.g. *no one help housework*, *no one to count for help*, *no financial help acquired*, *unsatisfied house condition*, *noisy living environment*, *regular alcohol consumption*, did not show a risk to survival, likely explained by the following. First, individuals who lacked specific social support (e.g. *help with housework*) were capable of looking after themselves, suggesting a better health status and lower mortality risk. Certain variables had different meanings as interpreted: e.g. *consume alcohol regularly* did not mean alcohol abuse but referred to a healthy state of alcohol consumption. The negative AR value for women in the age group 65+ indicates that at advanced ages drinking alcohol should not be encouraged in women. Also, such a univariable analysis tends to overlook the interplay of multiple protective factors. These findings are compatible with previous research.^7^

Consistent with the previous studies,^22^, ^33^ our data showed that the relative heterogeneity of frailty declined with age and with increased deficit accumulation respectively. More interestingly, women in this study showed a lower level of the relative heterogeneity of frailty than did men in any given age group. A higher survival rate is associated with a higher level of the relative heterogeneity of frailty. This observation is related to the one that women had lower mortality rates than men in the same degree of frailty heterogeneity. Given that variety in the response repertoire in Ashby's law of “requisite variety”^16^ can be well represented by the coefficient of variation,^22^ the results indicate that with age women tend to lose more varieties in the response repertoire than men; meanwhile, the remaining varieties in women seem to be more effectively mount overcome the insults. Therefore, compared with men in the same age group, frailer women having more effective responses retained to fight over come the environmental insults to maintain the systems' integrity. This difference might also partially explain the higher health expectancy for women.

Extrinsic protective factors can benefit the maintenance of functional health status and even reduce mortality.^23^ This statement is supported by the study that the summative effect of protective factors can increase in the relative heterogeneity of frailty in different age groups resulting in a lower mortality rate. Such protective effect appeared to be diminished after the age of 75, suggesting that when a body system comes closer to redundant exhaustion, extrinsic protective factors become less effective, while the intrinsic factors become more effective for an individual to overcome perturbation and maintain homeostasis. The measurable parameters such as coefficient of variation, which might represent intrinsic factors can provide further insights. This understanding is helpful for public health planning and clinical decision making to better care for older patients.

Our study has several limitations. First, all the variables used to construct FI and PI were based on self‐reported data, the accuracy of which might not be the same as a clinical assessment. Future work on frailty heterogeneity considering gender disparity in clinical settings will be of particular interest. Also, our dataset was based on the health measures dating from 1992, which are likely different from the current situation in China. Over the past two decades, fast economic growth in China has brought dramatic changes in education, income, lifestyle, culture, and health care, which is likely to influence the level of the health measures^6^ and thus the levels of FI and PI of the population. There is a high demand for health care improvement, for which continued efforts are being made.^37^ Despite the limitations, our study has demonstrated consistent results such as coefficient of variations, FI and PI estimates, their relationship with age, sex disparity and the association of those estimates, consistent with previous publications.^1^, ^12^, ^13^, ^34^, ^35^, ^36^, ^38^

In conclusion, our data suggest that a gender difference exists in the relative frailty heterogeneity measured by the coefficient of variation of the frailty index. Having more health‐protective factors can help decrease deficit accumulation and increase relative frailty heterogeneity among older adults, and be associated with reduced mortality rates. This finding suggests the interplay of intrinsic and extrinsic factors in determining health outcomes, which can provide insights for promoting public health in aging.

## CONFLICTS OF INTEREST

Nothing to disclose.

## AUTHOR CONTRIBUTIONS

ZY processed and analyzed the data, prepared the result presentation, and drafted the first manuscript. CW and ZT provided the dataset, helped process the data, and reviewed and edited the manuscript. XS conceptualized the research question and design, reviewed the data analysis and result presentation, and revised the first manuscript draft. All authors contributed to the study finding impartation, edited the various versions of the manuscript, and agreed upon publication of the paper.

## References

[1] Mitnitski A , Song X , Rockwood K . Improvement and decline in health status from late middle age: modeling age‐related changes in deficit accumulation. Exp Gerontol. 2007;42:1109‐1115. 17855035 10.1016/j.exger.2007.08.002

[2] Rockwood K , Mitnitski A . Frailty defined by deficit accumulation and geriatric medicine defined by frailty. Clin Geriatr Med. 2011;27:17‐26. 21093719 10.1016/j.cger.2010.08.008

[3] Fallah N , Mitnitski A , Searle SD , et al. Transitions in frailty status in older adults in relation to mobility: A multistate modeling approach employing a deficit count. J Am Geriatr Soc. 2011;59:524‐529. 21391943 10.1111/j.1532-5415.2011.03300.x PMC3125634

[4] Fried LP , Tangen CM , Walston J , et al. Frailty in older adults: evidence for a phenotype. J Gerontol A Biol Sci Med Sci. 2001;56:M146‐M156. 11253156 10.1093/gerona/56.3.m146

[5] Vaupel JW , Carey JR , Christensen K , et al. Biodemographic trajectories of longevity. Science. 1998;280:855‐860. 9599158 10.1126/science.280.5365.855

[6] Morley JE , Perry M III , Miller DK . Something about frailty. J Gerontol Med Sci. 2002;57:698‐704. 10.1093/gerona/57.11.m698 12403796

[7] Woo J , Goggins W , Sham A , Ho SC . Social determinants of frailty. Gerontology. 2005;51:402‐408. 16299422 10.1159/000088705

[8] Mitnitski AB , Graham JE , Mogilner AJ , Rockwood K . Frailty and late life mortality in relation to chronological and biological age. BMC Geriatrics. 2002;2(1):1. 11897015 10.1186/1471-2318-2-1 PMC88955

[9] Woo J , Tang NL , Suen E , Leung JC , Leung PC . Telomeres and frailty. Mech Ageing Dev. 2008;129:642‐648. 18809425 10.1016/j.mad.2008.08.003

[10] Gavrilov L , Gavrilova N . The reliability theory of aging and longevity. J Theor BIiol. 2001;213:527‐545. 10.1006/jtbi.2001.2430 11742523

[11] Rockwood K , Rockwood MR , Mitnistski A . Physiological redundancy in older adults in relation to the change with age in the slope of a frailty index. J Am Geriatr Soc. 2010;58:318‐323. 20370858 10.1111/j.1532-5415.2009.02667.x

[12] Rockwood K , Mitnitski A . Limits to deficit accumulation in elderly people. Mech Ageing Dev. 2006;127:494‐496. 16487992 10.1016/j.mad.2006.01.002

[13] Bennett S , Song X , Mitnistiski A , Rockwood K . A limit to frailty in very old, community‐dwelling people: a secondary analysis of the Chinese longitudinal health and longevity study. Age Ageing. 2013;42:372‐377. 23232936 10.1093/ageing/afs180

[14] Shi J , Yang Z , Song X , et al. Sex differences in the limit to deficit accumulation in late middle‐aged and older Chinese people: results from the Beijing Longitudinal Study of Aging. J Gerontol A Biol Sci Med Sci. 2014;69:702‐709. 24127426 10.1093/gerona/glt143 PMC4022096

[15] Vardhan R , Seplaki XQL , Bandeen‐Roche K , Fried LP . Stimulus‐response paradigm for characterizing the loss of resilience in homeostatic regulation associated with frailty. Mech Ageing Dev. 2008;129:666‐670. 18938195 10.1016/j.mad.2008.09.013 PMC2650618

[16] Ashby WR . An Introduction to Cybernetics. 2nd ed. London, UK: Chapman & Hall Ltd; 1952:195‐218.

[17] Thaler DS . Design for an aging brain. Neurobiol Ageing. 2002;23:13‐15. 10.1016/s0197-4580(01)00262-7 11755011

[18] Champion EW , Jette A , Beckman B . An interdisciplinary geriatrics consultation service: a controlled trial. J Am Geriatr Soc. 1983;31:792‐796. 6361104 10.1111/j.1532-5415.1983.tb03401.x

[19] Becker PM , McVey LJ , Saltz CC , et al. Hospital‐acquired complications in a randomized controlled clinical trial of a geriatric consultation team. JAMA. 1987;257:2313‐2317. 3553627

[20] Applegate WB , Miller ST , Graney MJ , et al. A randomized, controlled trial of a geriatric assessment unit in a community rehabilitation hospital. N Engl J Med. 1990;322:1572‐1578. 2186276 10.1056/NEJM199005313222205

[21] Winograd CH . Targeting strategies: an overview of criteria and outcomes. J Am Geriatr Soc. 1991;39S:25‐35. 10.1111/j.1532-5415.1991.tb05930.x 1885875

[22] Mitnitski A , Rockwood K . Decrease in the relative heterogeneity of health with age. Mech Ageing Dev. 2006;127:70‐72. 16257034 10.1016/j.mad.2005.09.007

[23] Wang C , Song X , Mitnistski A , et al. Effect of health protective factors on health deficit accumulation and mortality risk in older adults in the Beijing Longitudinal study of aging. J Am Geriatr Soc. 2014;62:821‐828. 24749784 10.1111/jgs.12792

[24] Jiang J , Tang Z , Meng XJ , Futatsuka M . Demographic determinants for change in activities of daily living: a cohort study of the elderly people in Beijing. J Epidemiol. 2002;12:280‐286. 12164333 10.2188/jea.12.280 PMC10499483

[25] Tang Z , Wang HX , Meng C , et al. The prevalence of functional disability in activities of daily living and instrumental activities of daily living among elderly Beijing Chinese. Arch Geronto Geriatr. 1999;29:115‐125. 10.1016/s0167-4943(99)00026-6 15374065

[26] Shi J , Song X , Yu P , et al. Analysis of frailty and survival from late middle age in the Beijing longitudinal study of aging. BMC Geriatrics. 2011;11:17‐24. 21507234 10.1186/1471-2318-11-17 PMC3239314

[27] Yu P , Song X , Shi J , et al. Frailty and survival of older Chinese adults in urban and rural areas: results from the Beijing Longitudinal Study of Aging. Arch Gerontol Geriatr. 2012;54:3‐8. 21621282 10.1016/j.archger.2011.04.020

[28] Shenkin S , et al. Systematic reviews: guidance relevant for studies of older people. Age Ageing. 2017;46(5):722‐728. 28655142 10.1093/ageing/afx105 PMC5860219

[29] Mitnitski A , Song X , et al. Relative fitness and frailty of elderly men and women in developed countries and their relationship with mortality. J Am Geriatr Soc. 2005;53:2184‐2189. 16398907 10.1111/j.1532-5415.2005.00506.x

[30] Song X , Mitnitski A , Rockwood K . Prevalence and 10‐year outcomes of frailty in older adults in relation to deficit accumulation. J Am Geriatrics Soc. 2010;58:681‐687. 10.1111/j.1532-5415.2010.02764.x 20345864

[31] Searle SD , Mitnitski A , Gahbauer EA , Gill TM , Rockwood K . A standard procedure for creating a frailty index. BMC Geriatr. 2008;8:24. 18826625 10.1186/1471-2318-8-24 PMC2573877

[32] Kelsey JL , Whittemore AS , Evans AS , Thompson DW . Methods in Observational Epidemiology. 2nd ed. Oxford, UK: Oxford University Press; 1996.

[33] Rockwood K , Mogilner A , Mitnitski A . Changes with ages in the distribution of a frailty index. Mech Ageing Dev. 2004;125:517‐519. 15246748 10.1016/j.mad.2004.05.003

[34] Mitnitski AB , Song X , Rockwood K . The estimation of relative fitness and frailty in community‐dwelling older adults using self‐report data. J Gerontol A Biol Sci Med Sci. 2004;59:M627‐M632. 15215283 10.1093/gerona/59.6.m627

[35] Graham JE , Snih SA , Berges IM , Ray LA , Markides KS , Ottenbacher KJ . Frailty and 10‐year mortality in community‐living Mexican American older adults. Gerontology. 2005;55:644‐651. 10.1159/000235653 PMC2783319 19690395

[36] Song X , Mitnitski A , Rockwood K . Prevalence and 10‐year outcomes of frailty in older adults in relation to deficit accumulation. J Am Geriatr Soc. 2010;58:681‐687. 20345864 10.1111/j.1532-5415.2010.02764.x

[37] Yip W , Hsiao WC . The Chinese health system at a crossroads: anew infusion of government funds has spared debate in China about how best to transform money into effective services. Health Aff. 2008;27(2):460‐468. 10.1377/hlthaff.27.2.460 18332503

[38] Mitnitski A , Song X , Skoog I , et al. Relative fitness and frailty of elderly men and women in developed countries and their relationship with mortality. J Am Geriatr Soc. 2005;53:2184‐2189. 16398907 10.1111/j.1532-5415.2005.00506.x

